# A J-shaped relationship between body mass index and the risk of elevated liver stiffness: a cross-sectional study

**DOI:** 10.1186/s40001-023-01543-3

**Published:** 2023-12-04

**Authors:** Yuwei Liu, Sheng Yuan, Jing Zuo, Sha Liu, Xiaoyan Tang, Xia Li, Dongai Yao, Yalei Jin

**Affiliations:** 1 Department of General Practice, Zhongnan Hospital of Wuhan University, Wuhan University, Wuhan, 430071 Hubei China; 2 Physical Examination Center, Zhongnan Hospital of Wuhan University, Wuhan University, Wuhan, 430071 Hubei China

**Keywords:** Body mass index, Liver stiffness measurement, Risk factor, Cross-sectional study

## Abstract

**Background:**

Liver stiffness (LS) is regarded as an indicator of the stages of liver fibrosis and liver cirrhosis. Numerous studies have investigated the relationship between body mass index (BMI) and LS; however, the conclusions remain controversial. In the current study, we utilized transient elastography (TE) technique, which could measure LS in a non-painful and noninvasive way, to explore the relationship between BMI and the risk of elevated LS in common community residents.

**Methods:**

5791 participants were included in the present study. To calculate BMI value, height and weight of the participants were carefully measured. Liver stiffness measurement (LSM) > 9.1 kPa was considered as a cutoff suggesting elevated LS. The relationship of BMI and risk of elevated LS was derived using generalized linear regression models, and the threshold effect was then analyzed by smooth curve fitting and segmented regression model.

**Results:**

Elevated LS was detected in 230 participants (3.97%) using the TE technique. After potential confounders were adjusted according to the individual’s demographic variables, underlying comorbidities and blood biochemical test results, we observed a J-shaped relationship between BMI and the risk of elevated LS, with the inflection point at 23.05 kg/m^2^. The effect size (and confidence interval) was 0.84 (0.71, 0.98) on the left side of the inflection point, and 1.32 (1.24, 1.41) on the right side of it.

**Conclusions:**

Our study found a novel J-shaped relationship between BMI and the risk of elevated LS assessed by TE technique. Abnormal BMI, either higher or lower, was associated with an increased risk of elevated LS.

## Background

Repeated liver injury secondary to any cause would lead to progressive fibrosis, and eventually resulting in liver cirrhosis [1]. Even though advanced liver cirrhosis could manifest in many systems, there is no specific clinical symptoms or signs indicating early stage liver fibrosis [2]. It has been reported that using the degree of liver fibrosis can predict adverse patient outcomes, and thus monitoring liver stiffness (LS) in the common community residents without advanced liver diseases may be a cost-effective way in the secondary and tertiary prevention for liver diseases [3].

Liver biopsy has always served as the golden standard for the diagnosis of liver fibrosis. However, this procedure is not only invasive and painful, but may also cause potentially life-threatening complications. Many studies reported that TE-based liver stiffness measurement could be used as a rapid, accurate and most importantly, non-invasive way to measure liver fibrosis and liver cirrhosis in people with or without diseases [4, 5]. Notably, the strong association between LSM values and the histological stages of liver fibrosis makes it an ideal alternative for liver biopsy [6, 7], especially in people with no or few symptoms.

Obesity, classified as BMI ≥ 30 kg/m^2^ in Caucasian populations, or BMI ≥ 25 kg/m^2^ in Asian–Pacific populations [8], is related to higher risk of advanced liver disease [9–11]. Several studies have explored the relationship between BMI and the risk of liver fibrosis [12, 13]; however, the conclusions remain controversial. Here, we took advantage of TE technique to measure LS in common community-based residents, and to further analyze the association between BMI and the risk of elevated LS in this population.

## Methods

### Study design

**Fig. 1:**
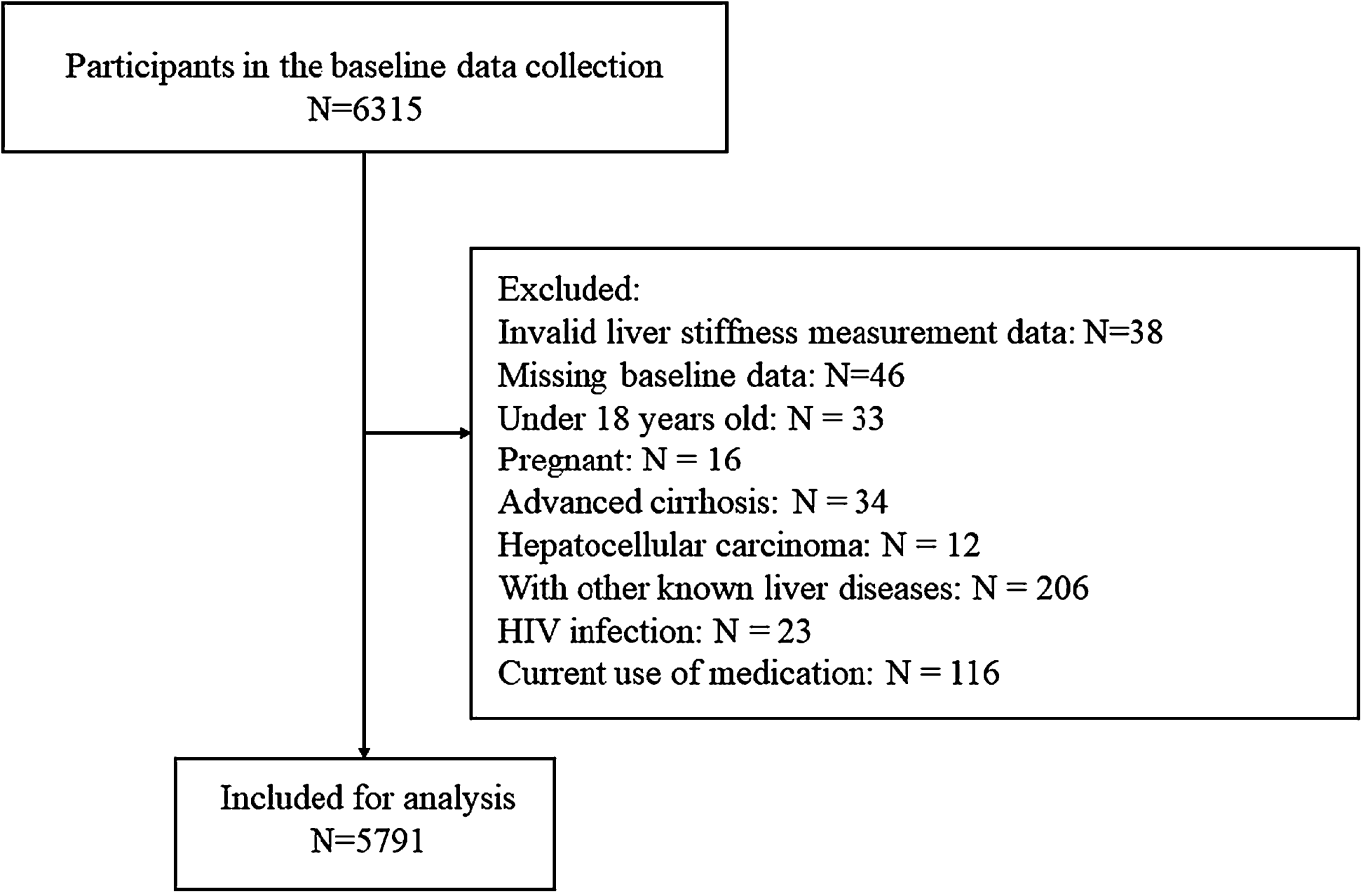
Study population

In this cross-sectional study, the inspected population was sequentially selected from community-dwelling residents who undertook annual medical examinations and LSM in Zhongnan Hospital of Wuhan University in the year of 2019 (from January 1, 2019, to December 31, 2019). Clinical data of 6315 participants were obtained. After running our exclusion criteria listed in Fig. 1, a total of 5791 participants were enrolled in the final analysis. Exclusion criteria were as follows: invalid liver stiffness measurement data, missing baseline data, age under 18 or pregnant, diagnosed with hepatocellular carcinoma or advanced liver cirrhosis (Child–Turcotte–Pugh B and C), other known liver disease (hepatitis B and C, etc.), heavy alcohol drinking [14] (alcohol intake > 30 g/d for males and > 20 g/d for females), human immunodeficiency virus infection, and current use of medication.

All procedures were conducted following the Helsinki Declaration of 1975, with revisions made in 2008, and were approved by the Ethics Committee of Zhongnan Hospital of Wuhan University (The Approval Number is 2021094 K).

### Clinical and laboratory data collection

The demographic features, comorbidities, laboratory findings and other relative information was accumulated from the hospital records. Data concerning co-morbidities were collected from the self-report questionnaire recording in the hospital records. Coronary heart disease (CHD) was defined as having been diagnosed in a medical institution with coronary angiography. Hypertension was defined as the use of antihypertensive agents or having been diagnosed in a medical institution. Diabetes was defined as receiving oral hypoglycemic agents or insulin treatment, or having been diagnosed in a medical institution. All blood samples were collected in the morning after an overnight fast and were processed within 2 h of collection. Blood Urea nitrogen (BUN), and creatinine were measured by enzymatic method. Alanine aminotransferase (ALT) and aspartate aminotransferase (AST) were measured by velocity method. Automated chemistry analyzer (Beckman Coulter chemistry analyzer AU5800 series, Tokyo, Japan) was used for measuring the levels of fasting blood–glucose (FBG), Uric acid, total cholesterol (TC), high-density lipoprotein cholesterol (HDL), low-density lipoprotein cholesterol (LDL) and triglycerides (TG). Two independent investigators were involved in reviewing medical records to ensure data accuracy.

BMI was computed on the basis of weight in kilograms and height in meters. All participants were divided into four different groups according to the calculated BMI. The categories of BMI were based on the epidemiology of obesity in China and defined as followed [15, 16]: underweight (BMI < 18.5 kg/m^2^), normal weight (BMI 18.5–23.9 kg/m^2^), overweight (BMI 24–27.9 kg/m^2^) and obese (BMI ≥ 28 kg/m^2^).

Fasting blood was collected from peripheral venous. To ensure sample consistency, laboratory tests and LSM were performed on the same date, within a time window of no more than 3 h.

### Liver stiffness measurement (LSM)

LSM was detected with a FibroScan device (Echosens, Paris, France) on the right lobe of the liver. Depends on the BMI level, a 3.5 MHz standard M probe or a 2.5 MHz XL probe were used to examine their LS value [17]. The characteristics, principle and procedure of TE have been described in detail previously [18]. Participants were also requested to an overnight fast before TE measurements. Participants who could not complete the test were excluded from the study. The ratio of the inter-quartile range (IQR) of LSM to the median (IQR/M), as an indicator of variability, was calculated. Only the measures with an IQR/M ratio of the LSM value < 0.3, a success rate of at least 60% and at least ten valid consecutive measurements were considered as reliable. LSM that did not meet these criteria of reliability was, therefore, excluded [19].

A strong correlation between LSM using TE and the stages and severity of liver fibrosis was observed in previous studies [20, 21]. The optimal LSM threshold for elevated LS varies in different populations and in people with various etiology of chronic liver diseases. A recent study reported that a TE cutoff of 9.1 kPa would be more accurate when diagnosing significant fibrosis (≥ F2) in both European and Asian populations [22]. Therefore, we adopted this value in the current study, and thus, a liver stiffness threshold larger than 9.1 kPa was categorized as elevated LS.

### Statistical analysis

While categorical variables were presented as frequencies and proportions, continuous variables were presented as mean ± SD or median and interquartile. To analyze the differences among the BMI classifications, we used the one-way ANOVA to assess normally distributed continuous variables, the Kruskal–Wallis test for skewed continuous variables and the Chi-squared test for categorical variables.

BMI were first treated as a categorical variable and then as a continuous variable in our analysis. The underweight category was the reference for BMI classifications. We used univariate and multivariate logistic regression models to evaluate the relationship between BMI and elevated LS. Thus, original and adjusted odds ratios (ORs) and corresponding 95% confidence intervals (CIs) were computed accordingly. Considering the clinical significance, we included age, gender, mean arterial pressure (MAP), history of coronary heart disease, diabetes, hypertension, ALT, FBG, creatinine, TG, TC and HDL in the multivariate adjusted models. We selected these variables on the basis of their associations with the outcomes of interest and changes in effect estimates by at least 10% as well. The generalized additive model (GAM) was adopted to identify the non-linear relationship. Once the non-linear correlation was detected, a two-piecewise linear regression model was performed to calculate the threshold effect in terms of the smoothing plot. The segmented regression model and likelihood ratio test (LRT) were used to explore the threshold effect [23]. *P* values less than 0.05 (two-sided) were considered statistically significant. All statistical analyses were performed using Empower(R) software (www.empowerstats. com, X&Y solutions, Inc., Boston, MA) and R software (http://www.R-project.org).

## Results

The average age of the 5,791 participants enrolled in the final analyses was 47.97 (± 12.59 SD) years, with 3662 (63.24%) being male. Among these 5791 participants, elevated LS was detected in 230 (3.97%). We first classified these participants according to their BMI into underweight (BMI < 18.5 kg/m^2^), normal weight (BMI 18.5 ~ 23.9 kg/m^2^), overweight (BMI 24.0 ~ 27.9 kg/m^2^) and obese (BMI > 28.0 kg/m^2^) groups. Then, we compared their demographic features, as well as clinical and biochemical test results in each group (Table 1). Significant differences were observed among these groups. We found that participants in the obese group are more prone to have comorbidities, such as hypertension, coronary heart disease, diabetes and dyslipidemia. These people also present with elevated MAP, FBG, as well as AST and AST levels, which indicate worse liver functions. To further clarify the correlations between BMI and liver damage, we then compared LS measured by TE in each group. We found that participants in the normal weight group had a lowest prevalence of 1.72% for elevated LS, while abnormal BMI, whether increased or decreased, was associated with higher risk of elevated LS (2.59% for underweight, 4.54% and 14.37% for overweight and obese, respectively).

**Table 1:** Baseline characteristics of participants according to the body mass index (BMI) classifications

Variable	Underweight (< 18.5 kg/m^2^)	Normal weight (18.5–23.9 kg/m^2^)	Overweight (24.0–27.9 kg/m^2^)	Obese (≥ 28 kg/m^2^)	*P* value
	*n* = 193	*n* = 2852	*n* = 2224	*n* = 522	
Age, years	40.56 ± 15.51	47.08 ± 12.73	49.93 ± 11.81	47.22 ± 12.21	< 0.001
Sex, n (%)					
Female	136 (70.47%)	1399 (49.05%)	504 (22.66%)	90 (17.24%)	< 0.001
Male	57 (29.53%)	1453 (50.95%)	1720 (77.34%)	432 (82.76%)	
Coronary heart disease, n (%)	2 (1.23%)	26 (1.24%)	42 (2.51%)	13 (3.55%)	0.003
Hypertension, n (%)	10 (6.17%)	166 (7.92%)	323 (19.28%)	90 (24.59%)	< 0.001
Diabetes, *n* (%)	0 (0.00%)	71 (3.39%)	84 (5.01%)	25 (6.83%)	< 0.001
MAP, mmHg	83.23 ± 11.19	88.46 ± 12.30	95.82 ± 12.64	100.09 ± 13.15	< 0.001
ALT, U/L	16.58 ± 13.61	21.59 ± 29.51	29.31 ± 20.54	39.66 ± 26.84	< 0.001
AST, U/L	22.58 ± 19.00	23.02 ± 14.44	25.47 ± 10.95	28.48 ± 13.26	< 0.001
FBG, mmol/L	5.10 ± 0.70	5.44 ± 1.11	5.77 ± 1.38	6.05 ± 1.55	< 0.001
Creatinine, μmol/L	61.68 ± 11.66	68.52 ± 18.81	74.91 ± 16.88	74.88 ± 14.21	< 0.001
Blood urea nitrogen, mmol/L	4.57 ± 1.22	4.91 ± 1.36	5.11 ± 1.27	5.12 ± 1.30	< 0.001
Uric acid, μmol/L	285.21 ± 69.94	330.92 ± 85.52	388.00 ± 91.61	421.58 ± 94.86	< 0.001
TG, mmol/L	0.98 ± 0.41	1.43 ± 1.05	2.05 ± 1.51	2.29 ± 1.70	< 0.001
TC, mmol/L	4.82 ± 0.97	5.12 ± 0.99	5.28 ± 1.06	5.24 ± 1.06	< 0.001
HDL, mmol/L	1.68 ± 0.37	1.45 ± 0.33	1.25 ± 0.28	1.17 ± 0.24	< 0.001
LDL, mmol/L	2.70 ± 0.77	3.09 ± 0.82	3.27 ± 0.89	3.29 ± 0.84	< 0.001
Incident of elevated liver stiffness, n (%)	5 (2.59%)	49 (1.72%)	101 (4.54%)	75 (14.37%)	< 0.001

Data are presented as mean ± SD, n (%), n/N (%), or median (IQR). *P* values comparing groups are from a *χ*^2^ test for categorical variables, and ANOVA for continuous variables

*MAP*   mean arterial pressure, *ALT*   alanine transaminase, *AST*   aspartate transaminase, *FBG*   fasting blood–glucose, *TG*   total triglyceride, *TC*   total cholesterol, *HDL*   high-density lipoprotein, *LDL*   low-density lipoprotein

To eliminate interference of different baseline variables and to distinguish the relationship between each of them and elevated LS, we first used univariate logistic regression models. The univariate analysis indicated that age, history of coronary heart disease, diabetes, hypertension, MAP, ALT, AST, FBG, creatinine and TG were positively correlated with the prevalence of elevated LS; however, female gender and HDL were negatively correlated with it (Table 2).

**Table 2:** Unadjusted associations between baseline characteristics and incident of elevated liver stiffness

Variable	Statistics	Odds ratio (95% CIs)	*P* value
Age, years	47.97 ± 12.59	1.04 (1.02, 1.05)	< 0.0001
Sex, *n* (%)			
Male	3662 (63.24%)	1.0	
Female	2129 (36.76%)	0.47 (0.34, 0.64)	< 0.0001
CHD, *n* (%)			
No	4216 (98.07%)	1.0	
Yes	83 (1.93%)	2.80 (1.38, 5.67)	0.0045
Diabetes, *n* (%)			
No	4119 (95.81%)	1.0	
Yes	180 (4.19%)	3.19 (1.97, 5.15)	< 0.0001
Hypertension, *n* (%)			
No	3710 (86.30%)	1.0	
Yes	589 (13.70%)	2.46 (1.75, 3.44)	< 0.0001
MAP, mmHg	92.16 ± 13.26	1.04 (1.03, 1.05)	< 0.0001
ALT, U/L	26.02 ± 26.36	1.02 (1.02, 1.03)	< 0.0001
AST, U/L	24.44 ± 13.40	1.04 (1.04, 1.05)	< 0.0001
FBG, mmol/L	5.61 ± 1.27	1.29 (1.21, 1.36)	< 0.0001
Creatinine, μmol/L	71.32 ± 17.87	1.01 (1.00, 1.01)	0.0149
TG, mmol/L	1.73 ± 1.35	1.19 (1.12, 1.26)	< 0.0001
TC, mmol/L	5.18 ± 1.03	1.05 (0.93, 1.19)	0.4433
HDL, mmol/L	1.35 ± 0.33	0.28 (0.18, 0.45)	< 0.0001
LDL, mmol/L	3.16 ± 0.86	1.00 (0.86, 1.17)	0.9827

Data are presented as mean ± SD, n (%), n/N (%), or median (IQR)

*CHD*   coronary heart disease, *MAP*   mean arterial pressure, *ALT*   alanine transaminase, *AST*   aspartate transaminase, *FBG*   fasting blood–glucose, TG   total triglyceride, *TC*   total cholesterol, *HDL*   high-density lipoprotein, *LDL*   low-density lipoprotein

The relationship between BMI and LS was further analyzed using logistic regression models. As shown in Table 3, these models were adjusted based on the participants’ demographic variables, underlying comorbidities and their blood biochemical test results. We noticed that with every unit of BMI increase, the prevalence of elevated LS increased by 29% (OR = 1.29; 95% CI 1.24–1.34; *P* < 0.0001) in the original, unadjusted model. Besides, compared to the underweight group, OR for the normal weight group (OR = 0.66; 95% CI 0.26–1.67; *P* = 0.3775) was lower, and ORs for the overweight (OR = 1.79; 95% CI 0.72–4.45; *P* = 0.2105) and obese groups (OR = 6.31; 95% CI 2.51–15.85; *P* < 0.0001) were higher. We then made partial adjustment to exclude the influence of demographic variables and underlying comorbidities of the participants in Model 1, and full adjustment by adding the baseline levels of ALT, FBG, Creatinine, TG and HDL in Model 2. Our analysis results showed that the correlation remains in this situation (OR = 1.29; 95% CI 1.23–1.36; *P* < 0.0001 for Model 1, OR = 1.23; 95% CI 1.16–1.30; *P* < 0.0001 for Model 2), which confirmed the relationship between BMI as a continuous variable and increased risk of elevated LS. Moreover, in the fully adjusted Model 2, the normal weight participants exhibited the lowest risk of elevated LS in comparison with the underweights and the obese participants, although the *P* values were not statistically significant.

**Table 3:** Risk associations between body mass index (BMI) and incident of elevated liver stiffness

	Unadjusted Odds ratio (95% CIs)	*P* value	Model 1^a^ Odds ratio (95% CIs)	*P* value	Model 2^b^ Odds ratio (95% CIs)	*P* value
BMI	1.29 (1.24, 1.34)	< 0.0001	1.29 (1.23, 1.36)	< 0.0001	1.23 (1.16, 1.30)	< 0.0001
BMI classifications						
Underweight	1.0		1.0		1.0	
Normal weight	0.66 (0.26, 1.67)	0.3775	0.51 (0.18, 1.48)	0.2152	0.47 (0.15, 1.41)	0.1755
Overweight	1.79 (0.72, 4.45)	0.2105	1.14 (0.40, 3.27)	0.8032	0.84 (0.28, 2.54)	0.7560
Obese	6.31 (2.51, 15.85)	< 0.0001	4.32 (1.47, 12.66)	0.0077	2.60 (0.83, 8.16)	0.1023

^a^Model 1 was adjusted for age, sex, mean arterial pressure, history of coronary heart disease, history of Diabetes, history of Hypertension

^b^Model 2 was adjusted for age, sex, mean arterial pressure, history of coronary heart disease, history of Diabetes, history of Hypertension, alanine transaminase (ALT), fasting blood–glucose (FBG), Creatinine, total triglyceride (TG), high-density lipoprotein (HDL)

**Fig. 2:**
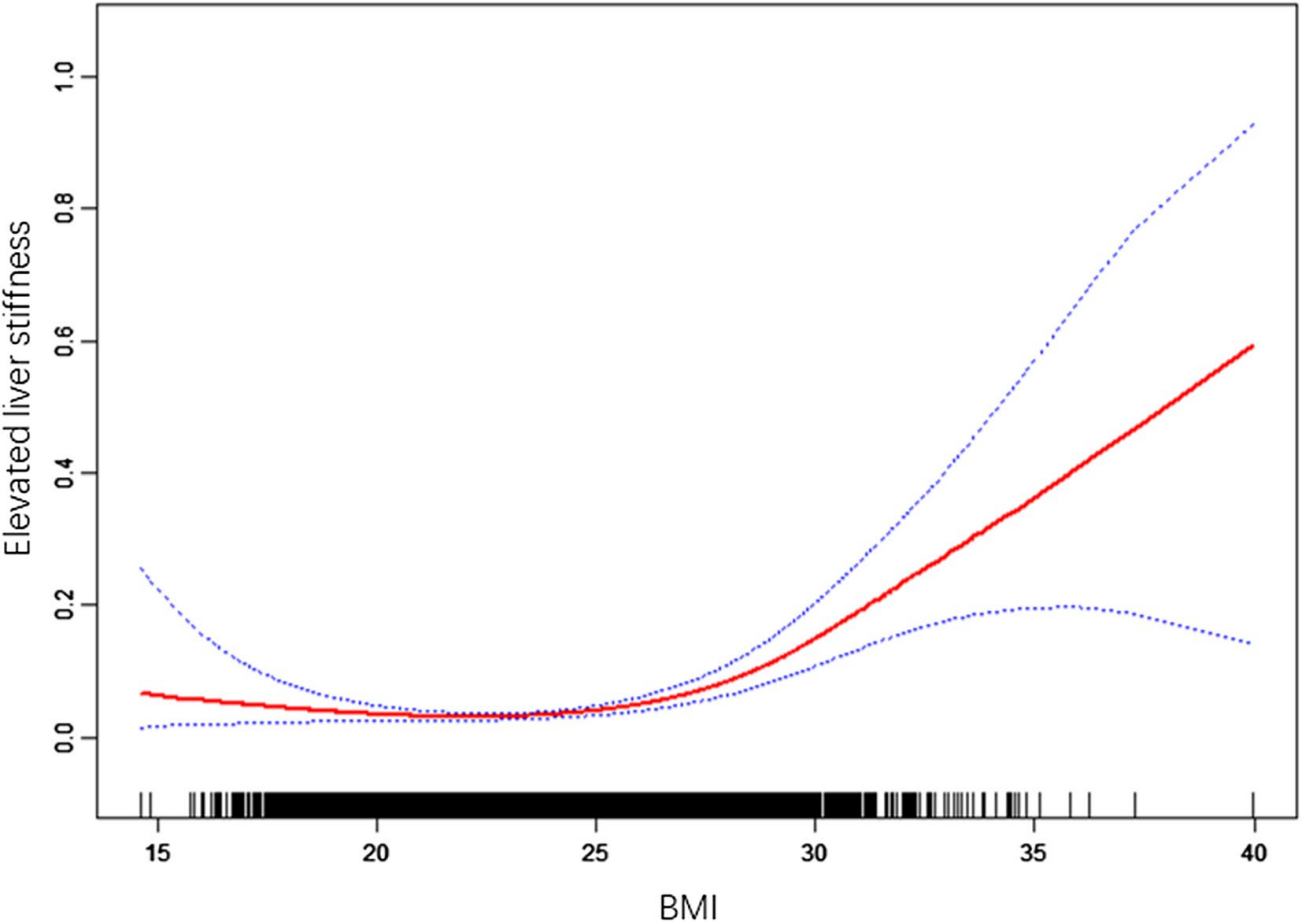
Smooth curve fitting for the relationship between body mass index (BMI) and incident of elevated liver stiffness. Each black band represents a sample. Solid red line represents the smooth curve fitting between variables. Blue bands represent the 95% of confidence interval from the fit

Since BMI is a continuous variable, the non-linear relationship should also be analyzed. Using spline smoothing fitting with adjustment of the previously mentioned demographic variables, underlying comorbidities and the participants’ blood biochemical test results, a J-shaped relationship between BMI and the prevalence of elevated LS clearly emerged (Fig. 2). Further analyses using a two-piecewise linear regression model revealed the inflection point was 23.05 kg/m^2^ (Table 4). Based on this J-shaped curve, to the left of the inflection point, per unit decrease of BMI is associated with 16% increase of the prevalence of elevated LS (adjusted OR = 0.84, 95% CI 0.71–0.98, *P* = 0.027); on the other side, to the right of the inflection point, per unit increase of BMI is associated with 32% increase of the prevalence of elevated LS (adjusted OR = 1.32, 95% CI 1.24–1.41, *P* < 0.0001).

**Table 4:** Threshold analysis for the relationship between body mass index (BMI) and incident of elevated liver stiffness

Models	Adjusted OR (95% CI)^a^	*P*
Model I		
One line slope	1.23 (1.16, 1.30)	< 0.0001
Model II		
Turning Point of BMI (K point)	23.05	
< K slope 1	0.84 (0.71, 0.98)	0.027
> K slope2	1.32 (1.24, 1.41)	< 0.0001
LRT test	< 0.001^b^	

^a^Adjusted for age, sex, mean arterial pressure, history of coronary heart disease, history of Diabetes, history of Hypertension, alanine transaminase (ALT), fasting blood–glucose (FBG), Creatinine, total triglyceride (TG), high-density lipoprotein (HDL)

^b^Means that Model II is significant different from Model I

LRT test means Logarithmic likelihood ratio test

## Discussion

In this study, we examined the relationship between BMI and the risk of elevated LS in community-based residents, using TE as our measurement tool, and observed a J-shaped curve between them. An inflection point was detected at 23.05 kg/m^2^, which is in the ‘normal BMI’ range. BMI seemed to have opposite influences on the risk of elevated LS on both sides of the inflection point. On the right side, BMI was positively associated with the risk of increased LS; however, a significantly negative association was found on the left side. That is to say, people with very low or very high BMI are more prone to present with elevated LS, while a normal BMI could be treated as a protective factor for liver function.

Previous researches have been conducted to detect the prevalence of liver fibrosis measured with TE in general populations. Two researches based on the Rotterdam Study, a European cohort, used LSM ≥ 8.0 kPa as their cutoff to suggest clinically relevant fibrosis [24, 25]. The use of a lower cutoff value would lead to a higher estimated prevalence of fibrosis in their studies (5.6% and 9.6%) in comparison with ours. When the cutoff value was raised to ≥ 9.0 kPa, Caballeria L et al. reported that the estimated prevalence of elevated liver stiffness was 3.6% [26], which is similar to our result of 3.97%. They have also suggested that the best cutoff of liver stiffness for significant liver fibrosis (F2–F4) should be 9.2 kPa, but that study was conducted in southwest Europe and did not include Asian participants. Recently, Serra-Burriel M et al. published their study result with much larger cohorts from Europe and Asia included [22]. They suggested using a TE cutoff of 9.1 kPa was more accurate to diagnose significant fibrosis (≥ F2) in the European and Asian populations. Therefore, we endorsed this value in our study.

Many studies have been devoted to address the association between BMI and the risk of elevated LS evaluated by TE in general population. Conti F, et al. explored factors influencing LSM in healthy population [27], which was based on the Bagnacavallo study. The study was a cross-sectional, community-based investigation among the dwellings of Bagnacavallo, Italy. Due to limited population in their study, participants with low and high BMI were both underrepresented, and unfortunately no significant correlation was observed. The Rotterdam Study conducted on another European cohort proved that there was no association between BMI and increase of LSM [24]. However, a study conducted in India argued that healthy subjects, either lean or obese, present with higher LSM values than those with normal BMI [28], which was in accordance with our finding of a J-shaped relationship between BMI and risk of elevated LS.

Even though debates about the influence of obesity on LSM have never stopped, studies indicated that obesity could act as a single or additive risk factor of elevated TE reading [29]. Especially in people with hepatitis B and C, life style modification with a purpose to reduce BMI should be encouraged to promote fibrosis improvement [4, 30, 31]. Unlike patients with hepatitis, in which LSM is widely used to indicate early stage of liver cirrhosis, the LSM of relatively healthy population has been overlooked. Moreover, the effect of underweight on LSM values in a healthy liver is yet unsolved. Large numbers of researches focusing on the effect factors of LSM were conducted in patients with liver diseases. The degree of hepatic fibrosis, central venous pressure, extrahepatic cholestasis and inflammation have been reported positively correlated with LSM values [32–35]. Individuals with the above conditions tended to have a lower BMI or be leanness as a result of chronic liver diseases. However, whether underweight would affect the LSM via the above mechanisms in the healthy population is remained to be explored. Researchers speculated that this may be due to different viscoelastic properties in the normal liver, where cellular components, cytoskeleton as well as Glisson’s capsule contributed more than collagen tissue to the exact LS value [28]. Further exploration into the biophysical properties of the liver would help to unravel the determinants of tissue elasticity at physiologic state in the future.

Here, we reported three major strengths in this study. First of all, although this was an observational study, which could be susceptible to various confounders, we managed to adopt hierarchical adjustments in the process of statistics to minimize residual confoundings. To do this, we not only selected the variables on the basis of their associations with the BMI and/or elevated LS, but also the ones changed in effect estimates of more than 10%. We found that compared with the crude regression analyses, the relationship remained unchanged after demographic and clinical variables were further adjusted. Second, we used both generalized linear model and generalized additive model to evaluate the linear and nonlinear relationship between BMI and the risk of elevated LS, respectively. GAM has been regarded as a persuasive way in the assessment of non-linear relations, As GAM can deal with non-parametric smoothing and fit regression splines to the data, the use of GAM could help us to discover the true relationships between exposures and outcomes. Finally, we evaluated the robustness of the findings by treating BMI both as a categorical and a continuous variable.

Some limitations should not be overpassed. First, liver biopsy is still widely accepted as the golden standard to diagnose liver fibrosis and liver cirrhosis, which could not be fully replaced by TE. However, studies have shown that elevated LS measured by TE could probably serve as an early predictor even before fibrosis was detected by liver biopsy [4–7]. With its rapid and non-invasive procedure, liver stiffness measurement has now been regarded as more cost-effective, applicable and potentially cost saving method to identify high-risk groups in the common residents. Second, an analytical cross-sectional study has its own limitations. Because it only provided weak evidence in analyzing the relationship between exposure and outcome and thus made it hard to differentiate between the cause and effect. Third, limited by raw data, we cannot elucidate the influence of waist-to-hip ratio and body weight changes on liver stiffness. There could still be some residual yet relevant confoundings needs to be considered. Finally, as all participants in this study were Chinese, it is not appropriate to apply the results directly to other ethnicities.

## Conclusions

BMI was independently related to the risk of elevated LS. The current study reported a novel J-shaped relationship between BMI and the incident of elevated LS measured by TE. A higher or lower BMI was associated with an increased risk of elevated LS, which is further associated with impaired liver function.

## Data Availability

The data that support the findings of this study are available on request from the corresponding author. The data are not publicly available due to privacy or ethical restrictions.

## Abbreviations

LS: Liver stiffness
BMI: Body mass index
TE: Transient elastography
LSM: Liver stiffness measurement
MAP: Mean arterial pressure
ALT: Alanine transaminase
AST: Aspartate transaminase
FBG: Fasting blood–glucose
TG: Total triglyceride
TC: Total cholesterol
HDL: High-density lipoprotein
LDL: Low-density lipoprotein
